# Closed Genome Sequences and Antimicrobial Resistance Profiles of Eight Wild Bird *Salmonella* Isolates Obtained with MinION and Illumina MiSeq Sequencing

**DOI:** 10.1128/MRA.00228-20

**Published:** 2020-06-18

**Authors:** Sohail Naushad, Marc-Olivier Duceppe, Andrée Ann Dupras, Ruimin Gao, Dele Ogunremi

**Affiliations:** a Ottawa Laboratory Fallowfield, Canadian Food Inspection Agency, Ottawa, Ontario, Canada; University of Maryland School of Medicine

## Abstract

Complete genome sequences of eight isolates of Salmonella enterica subsp. *enterica* from Canadian wild birds were determined by MinION and Illumina MiSeq sequencing. Assembled chromosomes had an average size of 4,833,662 bp. Salmonella enterica serovar Worthington obtained from partridge and quail carried 267-kb plasmids, which contained multiple antimicrobial resistance genes.

## ANNOUNCEMENT

Multidrug antimicrobial resistance (AMR) in *Salmonella* species is considered a global public health threat (1–3), and there is a need to develop microbial sequence resources to evaluate possible contributions by *Salmonella* strains of wild bird origin. We sequenced eight *Salmonella* organisms isolated from Canadian wild birds from the provinces of British Columbia, Ontario, Saskatchewan, and Newfoundland and Labrador. The organisms were isolated using a combination of primary enrichment culture in peptone broth at 37°C overnight, followed by inoculation in Rappaport-Vassiliadis selective enrichment broth at 42°C overnight, and plating onto XLT-4 selective agar to grow the bacterial colonies (Table 1). High-quality DNA was extracted from an overnight culture (1 ml of brain heart infusion medium at 37°C) using the Wizard genomic DNA purification kit (Promega, Madison, WI) and assessed with a spectrophotometer (DU 730; Beckman Coulter, Mississauga, ON, Canada) and a Qubit 2.0 fluorometer (Life Technologies, Carlsbad, CA). Library construction for Illumina MiSeq sequencing was carried out with the Nextera XT DNA kit (Illumina, San Diego, CA), and sequencing and read trimming were performed as described (4). Libraries for MinION sequencing were prepared without shearing using the 1D ligation sequencing kit (SQK-LSK108), and DNA was barcoded with the native barcoding expansion kit (EXP-NBD103) according to the manufacturer’s instructions (Oxford Nanopore Technologies, Inc., Oxford, UK). The final library was analyzed by MinION sequencing on a FLO-MIN106 (R9.4.1) flow cell for 48 h. Fast5 reads were basecalled using the high-accuracy basecalling algorithm in Guppy (v3.1.5), and the resulting fastq reads were trimmed with Porechop v0.2.3 (default settings) and filtered with Filtlong v0.2.0, keeping the top 90% quality reads or reads with 100× coverage. Hybrid assembly of Illumina paired-end reads and MinION reads was achieved with SPAdes v3.11.1 (5) and polished with Pilon v1.23 (6) using Unicycler v0.4.4 (7). Overlapping regions were trimmed and the genomes were rotated using the fixstart program in Circlator (8). The genomes were quality checked with QUAST v5.0.2 (9), and the depth of sequencing coverage was determined by mapping individual reads against the assembled genomes using minimap2 v2-2.17, with visualization using Qualimap v2.2.1. The presence of AMR genes was determined with ResFinder v3.0 (10). Default parameters were used for all analyses except where otherwise noted. Assembled genomes and raw reads were submitted to GenBank (Table 1). Each polished genome contained a single chromosome and frequently multiple contigs representing plasmid and/or phage sequences. Six isolates (ST-13, ST-29, ST-32, ST-33, ST-35, and ST-87) were identified as Salmonella enterica subsp. *enterica* serovar Typhimurium, while the remaining two isolates (SW-37 and SW-70, obtained from a partridge and a quail, respectively) were identified as Salmonella enterica subsp. *enterica* serovar Worthington. The average chromosome size was 4,833,662 bp. The large virulence plasmid of *S.* Typhimurium was found in isolates ST-29, ST-33, ST-35, and ST-87. Apart from the large plasmid, isolate ST-35 contained an additional 6,050-bp *Salmonella*-specific plasmid and a 95,814-bp sequence, identified as plasmid pEC006 from Escherichia coli by BLAST searching of the nucleotide database. Both *S*. Worthington isolates (SW-37 and SW-70) contained a very large 267-kb plasmid with AMR genes for aminoglycosides [*aph(3′)-Ia* and *aadA7*], tetracycline [*tet*(B)], and sulfonamides (*sul1*).

**TABLE 1:** Source information and genomic characteristics of Salmonella enterica subsp. *enterica* isolates from Canadian wild birds

Strain	Sample name	Source	Province^*a*^	Serovar	Median read length (bp) for:		No. of contigs	Chromosome size (bp)	Plasmid size(s) (bp)	Bacteriophage size (bp)	GC content (assembled molecule) (%)	Depth of coverage (×)	GenBank accession no. for whole molecule(s)	SRA accession no. for MiSeq/MinION reads
					MiSeq reads	MinION reads								
ST-13	OLF_FSR1_WB_Finch_ST-13	Finch	BC	Typhimurium	300	9,002	1	4,819,669	—^*b*^	—	52.15	99	CP051269	SRX7862684 /SRX7862698
ST-29	OLF-FSR1_WB_Gull_ST-29	Gull	SK	Typhimurium	295	3,916	3	4,879,541	94,038	41,384	52.19	59	CP051286, CP051287, CP051288	SRX7862685 / SRX7862699
ST-32	OLF-FSR1_WB_Gull_ST-32	Gull	ON	Typhimurium	300	2,823	2	4,920,253	—	41,336	52.14	52	CP051284, CP051285	SRX7862692 / SRX7862686
ST-33	OLF-FSR1_WB_Hawk_ST-33	Hawk	ON	Typhimurium	301	5,468	2	4,818,617	93,798	—	52.18	99	CP051267, CP051268	SRX7862693 / SRX7862687
ST-35	OLF-FSR1_WB_Junco_ST-35	Junco	BC	Typhimurium	300	4,312	5	4,820,281	99,574, 95,663, 6,035	45,661	52.13	98	CP051279, CP051280, CP051281, CP051282, CP051283	SRX7862694 / SRX7862688
ST-87	OLF-FSR1_WB_Sparrow_ST-87	Sparrow	NL	Typhimurium	301	5,311	3	4,697,589	92,921	41,335	51.95	65	CP051276, CP051277, CP051278	SRX7862695 / SRX7862689
SW-37	OLF-FSR1_WB_Partridge_SW-37	Partridge	BC	Worthington	283	4,336	3	4,759,747	267,109	41,874	51.86	95	CP051273, CP051274, CP051275	SRX7862696 / SRX7862690
SW-70	OLF-FSR1_WB_Quail_SW-70	Quail	BC	Worthington	300	3,440	3	4,857,600	267,106	42,910	52.14	98	CP051270, CP051271, CP051272	SRX7862697 / SRX7862691

a BC, British Columbia; SK, Saskatchewan; ON, Ontario; NL, Newfoundland and Labrador.

b −, absent.

### Data availability.

Raw reads and assembled genomes were submitted to GenBank under BioProject number PRJNA605433 and the accession numbers are provided in Table 1.
